# Efficacy of Low Dose Proton Pump Inhibitor-Based Therapy to Eradicate *Helicobacter pylori* in Patients with Subtotal Gastrectomy

**DOI:** 10.3390/jcm8111933

**Published:** 2019-11-10

**Authors:** Dongwoo Kim, Sung Woo Jung, Dong-won Lee, Chang Min Lee, Seung Young Kim, Jong Jin Hyun, Young Kul Jung, Ja Seol Koo, Hyung Joon Yim, Sang Woo Lee

**Affiliations:** 1 Department of Internal Medicine, Korea University Ansan Hospital, 123, Jeokgeum-ro, Danwon-gu, Ansan-si, Gyeonggi-do 15355, Korea; blustick@hanmail.net (D.K.);; 2 Department of Surgery, Korea University Ansan Hospital, 123, Jeokgeum-ro, Danwon-gu, Ansan-si, Gyeonggi-do 15355, Korea

**Keywords:** *Helicobacter pylori*, eradication, proton pump inhibitor

## Abstract

Proton pump inhibitor (PPI)-based therapy is standard to eradicate *Helicobacter pylori* (*H. pylori*). Gastric acidity is lowered after gastrectomy because of bile reflux and impaired mechanism of acid secretion. Therefore, low-dose PPI may be effective for *H. pylori* eradication in the remnant stomach after gastrectomy. In this study, we compared the efficacy of low-dose PPI with standard double-dose PPI to eradicate *H. pylori* in patients who underwent subtotal gastrectomy. A total of 145 patients who were treated for eradication after gastrectomy was analyzed. They were treated with PPI-based triple regimen (PPI, clarithromycin and amoxicillin) for 14 days. We compared the eradication rate in the low-dose PPI group (lansoprazole 15 mg once daily) with that in the standard double-dose PPI group (lansoprazole 30 mg twice daily). The *H. pylori* eradication rate was 79.1% in the low-dose PPI group and 85.3% in the standard double-dose group; the difference was not significant statistically (*p* = 0.357). In the multivariate analysis, low-dose PPI (odds ratio (OR) = 1.79, 95% confidence interval (CI), 0.68–4.69) was not associated with eradication failure, while Billroth II anastomosis (OR = 4.45, 85% CI, 1.23–16.2) was significantly associated with eradication failure. Low-dose PPI-based triple regimen was as effective as standard double-dose PPI-based regimen for *H. pylori* eradication in patients with subtotal gastrectomy. Further study is needed to confirm the effect of low-dose PPI on H. pylori eradication in patients with gastrectomy.

## 1. Introduction

*Helicobacter pylori* (*H. pylori*) infection is a significant risk factor of gastric cancer in humans [1]. Eradication of *H. pylori* prevents gastric cancer in patients who are infected and reduces the development of metachronous gastric cancer in patients treated with endoscopic resection for early gastric cancer [2,3]. However, the effect of *H. pylori* eradication on the gastric remnant after gastrectomy has not been clearly determined. Recently, the positive effect of *H. pylori* eradication therapy in patients who underwent subtotal gastrectomy, such as reducing development of pre-cancerous lesion, was reported [4]. The Asia-Pacific consensus guideline recommended *H. pylori* eradication in gastric cancer patients with distal gastrectomy state [5].

Triple therapy, consisting of a standard double-dose of proton pump inhibitor (PPI) plus two antibiotics such as amoxicillin and clarithromycin, is the standard first-line treatment regimen for *H. pylori* eradication in Korea [6,7]. This regimen is also known to be effective in patients with subtotal gastrectomy [8]. However, gastric surgery causes dramatic changes in the intra-gastric environment such as decreased gastric acidity and the survival of *H. pylori* is disadvantageous after gastrectomy, the necessity of standard double-dose PPI treatment in patients undergoing gastrectomy is questionable. To date, there have been no studies evaluating the eradication rate of *H. pylori* using triple regimen including low-dose PPI in patients underwent gastrectomy.

This study was aimed to evaluate the effect of low-dose PPI-based therapy on the eradication rate of *H. pylori* compared with standard double-dose PPI-based regimen and analyze the factors that affect eradication failure in patients with gastric cancer undergoing gastrectomy.

## 2. Materials and Methods

**Figure 1:**
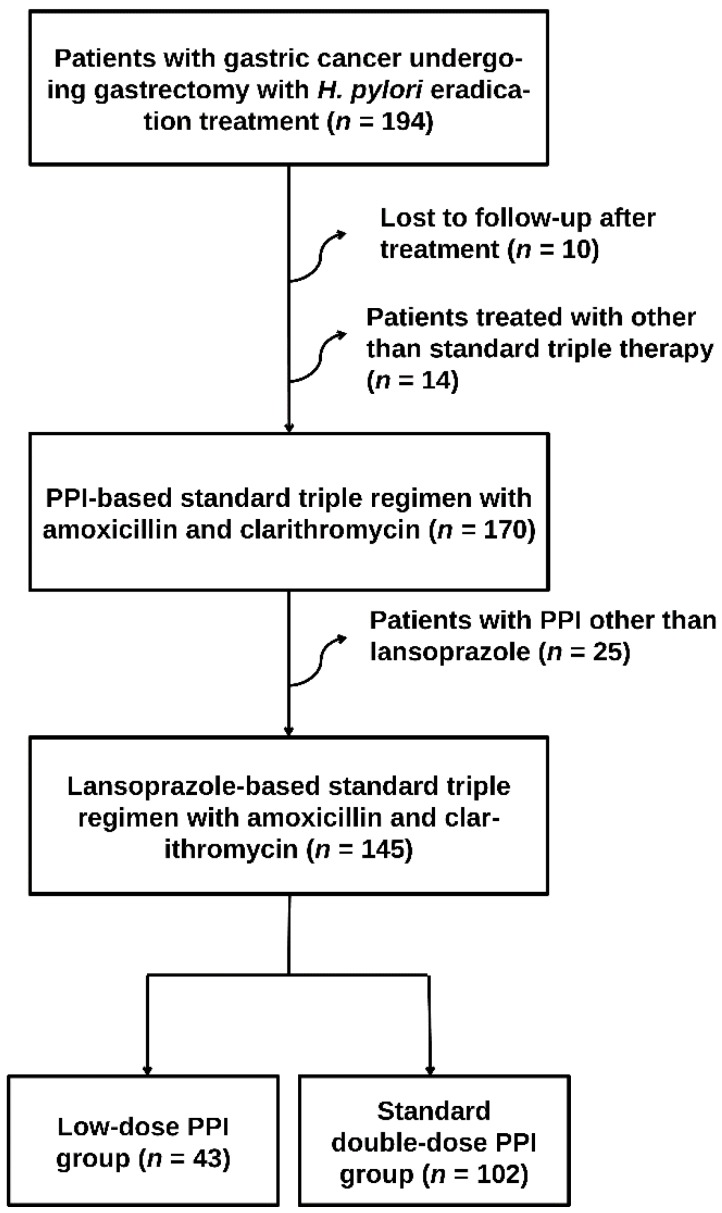
Flow diagram of the study population. Abbreviations: *H. pylori*, *Helicobacter pylori*; PPI, proton pump inhibitor.

In this retrospective study, we included all patients with gastric cancer who were treated for *H. pylori* eradication after gastrectomy between September 2008 and September 2017. A total of 145 patients were analyzed after excluding patients who were lost to follow-up, those who were treated with other than a standard triple regimen consisting of clarithromycin, amoxicillin and PPI, and those who used PPI other than lansoprazole (Figure 1). All patients who were diagnosed to have *H. pylori* infection were treated and the type of anastomosis was determined by surgeon according to the required extent of resection and the patient’s condition.

This study was approved by the institutional review board of the Korean University Ansan Hospital. The need for informed consent was waived in view of the retrospective study design.

### 2.1. Diagnosis of H. pylori Infection

Histologic examination was performed to confirm *H. pylori* infection using biopsy specimen taken from the cardia and fundus during annual surveillance endoscopy after gastrectomy. These specimens were fixed and stained with cresyl violet for microscopic examination. Patients were considered to be infected with *H. pylori* if the test was positive.

### 2.2. H. pylori Treatment and Detection of Eradication

Patients were treated with amoxicillin (1000 mg), clarithromycin (500 mg), and lansoprazole (15 mg) daily (low-dose PPI group) or lansoprazole (30 mg) twice daily (standard double-dose PPI group). These medications were administered for two weeks. To confirm *H. pylori* eradication, histologic examination was performed with a biopsy specimen taken during next follow-up surveillance endoscopy which was performed within 1 year after *H. pylori* eradication treatment. If *H. pylori* was not observed, it was considered eradicated.

### 2.3. Statistical Analysis

The value of continuous variables is expressed as the mean ± standard deviation (SD). Discrete or categorical variables are presented as a percentage. The groups were compared using the Student t-test for chi-square test. To evaluate risk factors for *H. pylori* eradication failure, univariate and multivariate logistic regression analysis was used to calculate the odds ratio (ORs) with 95% confidence intervals (95% CI). Covariates used in the multivariate analysis included variables with a significant result on the univariate analysis (*p* < 0.100), in addition to risk factors associated with eradication failure from a previous study and clinical experience [9,10]. Statistical analysis was performed using SPSS (version 23.0 for Windows, Chicago, IL, USA). All tests were 2-tailed, and a *p*-value of <0.05 was considered statistically significant.

## 3. Results

A total of 145 patients were analyzed. Of these, 43 patients were assigned to the low-dose PPI group and 102 patients to the standard double-dose PPI group. The mean age of patients was 56.9 ± 11.7 years. There were 32 (74.4%) men in the low-dose PPI group and 70 (68.6%) in the standard double-dose PPI group; men were more prevalent in both groups. There was no significant difference in baseline characteristics between the two groups in terms of age, sex, medical history, smoking and drinking status, cancer type, cancer histology (Lauren’s classification) and surgical method (Table 1).

**Figure 2:**
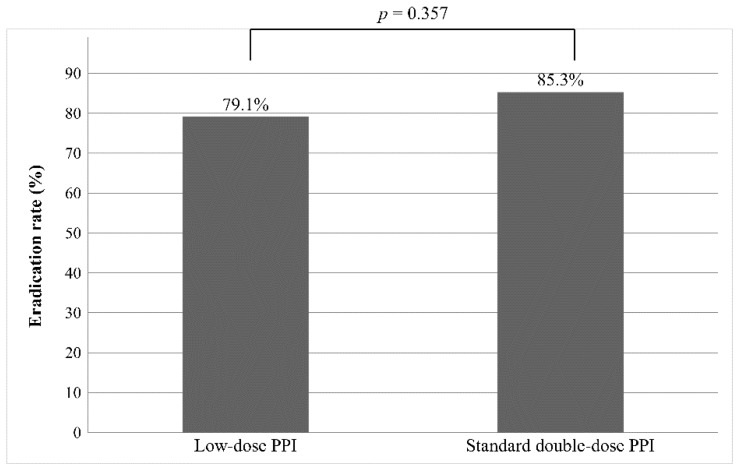
The eradication rate of *H. pylori* in low-dose and standard double-dose PPI group. Abbreviations: *H. pylori*, *Helicobacter pylori*; PPI, proton pump inhibitor.

After treatment with PPI-based triple therapy, the eradication rate in all patents was 84.3%. In particular, it was 79.1% in the low-dose PPI group and 85.3% in the standard double-dose PPI group. However, there was no significant difference (*p* = 0.357) in the eradication rate between the two groups (Figure 2).

Clinical factors associated with *H. pylori* eradication failure and their calculated ORs are summarized in Table 2. Univariate analysis showed that Billroth II anastomosis had approximately 4-fold higher risk of *H. pylori* eradication failure, compared with Billroth I anastomosis (OR = 4.45; 95% CI, 1.23–16.2). In the multivariate analysis, which was adjusted for clinical factors such as age and sex (Table 3), Billroth II anastomosis was the only clinical factors associated with *H. pylori* eradication failure (OR = 4.45; 95% CI 1.23–16.2). However, low-dose PPI was not significantly associated with *H. pylori* eradication failure (OR = 1.79, 95% CI 0.68–4.69).

## 4. Discussion

In this study, we demonstrated that the *H. pylori* eradication rate of low-dose PPI-based triple therapy was 79.1%. This percentage was not inferior to that of standard double-dose PPI-based regimen in patients with subtotal gastrectomy. Furthermore, low-dose PPI was not a significant risk factor for eradication failure in patients with gastrectomy.

Current treatments of *H. pylori* infection rely on acid suppression via PPI in combination with at least two antibiotics. The role of PPI in eradication therapy has been attributed to direct antibacterial activity and facilitation of increased antibiotics stability in the less acidic intra-gastric environment [11,12,13]. Also, since *H. pylori* replicates best at neutral pH, and antibiotics used for eradication such as amoxicillin and clarithromycin have a bactericidal effect to actively dividing bacteria only, a sufficient dose of PPI to raise intra-gastric pH is necessary to allow growth of *H. pylori* and increase the efficacy of growth-dependent antibiotics [14,15]. Based on previous studies reporting the low eradication rate of single dose PPI based regimens, standard double-dose PPI is considered the standard regimen for *H. pylori* eradication therapy [7].

Gastric surgery dramatically alters the intra-gastric environment. Studies have shown that the pH of intra-gastric juice becomes elevated due to increased bile reflux and decreased acid secretion arising from vagotomy [16,17]. Therefore, the rationale for standard double-dose PPI-based triple therapy is lacking in patients with gastrectomy, although it is known to be effective [8]. We found that a surgeon in our hospital inadvertently prescribed low-dose PPI for the treatment of *H. pylori* after subtotal gastrectomy. Because the increased intra-gastric pH suggested that low-dose PPI might have been effective, we analyzed these patients retrospectively and found that the eradication rate of the low-dose PPI-based regimen was approximately 80%; it was not inferior to that of the standard double-dose PPI regimen, which suggests that a low-dose PPI-based regimen seems to be effective in patients with high intra-gastric pH, such as seen after gastrectomy. Further studies evaluating the relationship between changes in intra-gastric acidity based on PPI dose and eradication rate in patients with gastrectomy are needed to better understand the role of low-dose PPI.

In addition, gastric resection and anastomosis method can change the pharmacokinetics of drugs, which may affect the efficacy of *H. pylori* eradication [18]. Onoda et al. reported a lower prevalence of *H. pylori* infection in patients who underwent gastrectomy with Billroth II anastomosis [19]. This was thought to reflect the role of bile reflux. However, in our study, Billroth II anastomosis had a 4.5 times higher risk for *H. pylori* eradication failure, compared with Billroth I anastomosis. Previous studies reported slower gastric emptying in patients with Billroth I anastomosis than in patients with Billroth II anastomosis [20,21]. Considering that local delivery of therapeutic agent was enhanced when gastric emptying was slow, gastric emptying delay associated with Billroth I anastomosis might have a positive effect on *H. pylori* eradication, which supports our results [22]. Although we cannot fully explain the reason for the difference of eradication rate according to anastomosis type in present study, changes in the drug absorption rate and metabolism, which may differ depending on the surgical method, are thought to influence the efficacy of *H. pylori* eradication therapy [23].

We acknowledge that our study has limitations. First, it was conducted at a single center and included a small number of patients retrospectively; our study focused mainly on patients who were prescribed low-dose PPI due to surgeon’s mistake at the time of *H. pylori* eradication, so the total number of patients was limited. As it is not a well-designed or statistically calculated prospective study, its results may not fully reflect the entire gastrectomy population. However, this is the first study using low-dose PPI in patients who underwent gastrectomy and it can be a basis for a future prospective study. Second, significant clinical information such as drug compliance, antibiotic resistance of *H. pylori* or the CYP2C19 status of the patients which has a significant impact on *H. pylori* eradication or adverse event after *H. pylori* eradication was not available. This is also a limitation of our retrospective study design. Therefore, a large scale prospective study is warranted to understand the impact of low-dose PPI on *H. pylori* eradication in patients who underwent gastrectomy more clearly.

## 5. Conclusions

In this study, we demonstrated that low-dose PPI-based regimen was as effective as standard double-dose PPI-based regimen for the eradication of *H. pylori* in the patients who underwent gastrectomy. Clinically, this PPI dose reduction would help to reduce the side effects of PPI as well as overall medical costs. Based on these results, a large-scale prospective study is needed to confirm the effect of low-dose PPI on *H. pylori* eradication in patients with gastrectomy.

## Figures and Tables

**Table 1:** Baseline characteristics of patients.

	Low-Dose PPI (*n* = 43) N (%)	Standard Double-Dose PPI (*n* = 102) N (%)	Total (*n* = 145) N (%)	*p*-Value
Age, mean ± SD	56.2 ± 11.4	57. 2 ± 11.9	56.9 ± 11.7	0.635
Male	32 (74.4)	70 (68.6)	102 (70.3)	0.487
DM	8 (18.6)	16 (15.7)	24 (16.6)	0.667
HTN	11 (25.6)	30 (29.4)	41 (28.3)	0.641
Alcohol	22 (51.2)	49 (48.0)	71 (49.0)	0.355
Smoking	14 (32.6)	42 (41.2)	56 (38.6)	0.994
Cancer type				0.828
EGC	30 (69.8)	73 (71.6)	103 (71.0)	
AGC	13 (30.2)	29 (28.4)	42 (29.0)	
Cancer histology (Lauren’s classification)				0.470
Intestinal type	18 (41.9%)	50 (49.0%)	68 (46.9%)	
Diffuse type	25 (58.1%)	52 (51.0%)	77 (53.1%)	
Anastomosis				0.595
Billroth I	15 (34.9)	32 (31.4)	47 (32.4)	
Billroth II	17 (39.5)	56 (54.9)	73 (50.3)	
Roux-en-Y	11 (25.6)	14 (13.7)	25 (17.2)	
Eradication rate, %	79.1	85.3	83.4	0.357

Abbreviations: PPI, proton pump inhibitor; DM, diabetes mellitus; HTN, hypertension; EGC, early gastric cancer; AGC, advanced gastric cancer.

**Table 2:** Univariate analysis of risk factors associated with Helicobacter pylori eradication failure.

Variates	Odds Ratio (95% CI)	*p*-Value
Age	1.02 (0.99–1.06)	0.215
Male	1.74 (0.60–5.00)	0.305
DM	1.41 (0.47–4.25)	0.538
HTN	1.33 (0.52–3.41)	0.548
Alcohol	1.28 (0.53–3.09)	0.577
Smoking	0.76 (0.30–1.92)	0.561
Cancer status		
EGC	1	
AGC	1.28 (0.50–3.26)	0.606
Cancer histology		
Intestinal type	1	
Diffuse type	1.05 (0.44–2.53)	0.909
Anastomosis		
Billroth I	1	
Billroth II	4.45 (1.23–16.2)	0.023*
Roux-en-Y	2.79 (0.57–13.6)	0.204
Use of low dose PPI	1.54 (0.61–3.84)	0.359

Abbreviations: CI, confidence interval; PPI, proton pump inhibitor; DM, diabetes mellitus; HTN, hypertension; EGC, early gastric cancer; AGC, advanced gastric cancer.

**Table 3:** Multivariate analysis of risk factors associated with Helicobacter pylori eradication failure.

Variates	Odds Ratio (95% CI)	*p*-Value
Age	1.02 (0.98–1.06)	0.255
Male	1.20 (0.35–3.99)	0.769
Anastomosis		
Billroth I	1	
Billroth II	4.45 (1.23–16.2)	0.023*
Roux-en-Y	2.79 (0.57–13.6)	0.204
Use of low dose PPI	1.79 (0.68–4.69)	0.235

Abbreviations: CI, confidence interval; PPI, proton pump inhibitor.
